# Retos y oportunidades en el estudio de vesículas extracelulares: contexto institucional a nivel mundial y situación actual en Colombia

**DOI:** 10.7705/biomedica.5749

**Published:** 2021-09-22

**Authors:** Susana Novoa-Herrán

**Affiliations:** 1 Grupo de Fisiología Molecular, Instituto Nacional de Salud, Bogotá, D.C., Colombia Grupo de Fisiología Molecular Instituto Nacional de Salud BogotáD.C Colombia

**Keywords:** vesículas extracelulares, exosomas, micropartículas derivadas de células, fenómenos químicos, técnicas de química analítica, terminología como asunto, guía de recursos, Extracellular vesicles, exosomes, cell-derived microparticles, chemical phenomena, chemistry techniques, analytical, terminology as topic, resource guide

## Abstract

En la última década se ha incrementado el número de estudios y publicaciones sobre las vesículas extracelulares y los exosomas. En Colombia, ha habido interés y avances en su estudio, lo que se evidencia en el aumento de publicaciones y proyectos de investigación. Sin embargo, este es un campo de investigación aún en desarrollo, con desafíos analíticos y limitaciones técnicas, por lo cual, en el planteamiento de los proyectos de investigación y desarrollo, es necesario considerar cuál es el estado del campo científico a nivel mundial en cuanto a la nomenclatura y la clasificación de las vesículas extracelulares, las técnicas, recursos, requisitos y especificaciones de calidad y las instituciones que regulan el campo. La respuesta a esta pregunta permitirá desarrollar estudios que cumplan con los estándares internacionales, y las exigencias y recomendaciones institucionales. Sin embargo, la información científica disponible se encuentra dispersa y no todos los aspectos son tratados a cabalidad.

En este actualización se condensa la información disponible y se presentan los términos oficiales para denominar las vesículas extracelulares y la nomenclatura aceptada actualmente, así como la evolución del campo, la homogenización de los parámetros experimentales, el establecimiento de autoridades científicas, instituciones y recursos, y las recomendaciones que se han generado a nivel mundial para el desarrollo de investigaciones en vesículas extracelulares, incluidos su aislamiento, caracterización y estudio funcional. Por último, se analiza el contexto nacional de una forma crítica, teniendo en cuenta las fortalezas institucionales, los errores usualmente cometidos, y las técnicas y tecnologías analíticas disponibles.

## Abstract (en)

In the last decade, the number of studies and publications on extracellular vesicles (EV) and exosomes has boomed. Colombia has displayed interest and progress in their study as shown in the increase of research project publications and products. However, this research field is still developing and has its own analytical challenges and technical limitations. For planning research projects and developing EV studies it is necessary to consider what is the state of the scientific field worldwide concerning EV nomenclature and classification, available techniques, resources, requirements and quality specifications, and the institutions that regulate the field. Answering this question will elicit EV studies that comply with international standards and respond to institutional demands and recommendations. However, the scientific information available is scattered and not all the aspects are considered in full.

In this update, the available information is condensed and the official terms and currently defined nomenclature is presented, as well as the evolution of the field, the homogenization of the experimental parameters, the establishment of scientific authorities, institutions, and resources, and the recommendations generated worldwide for their development and research including their isolation, characterization, and functional studies. Finally, I analyzed the national context in a critical way, considering institutional strengths, common mistakes, and available analytical techniques and technologies.

La comunicación entre las células y la capacidad que tienen de afectarse unas a otras caracteriza tanto a los organismos unicelulares como a los multicelulares, y es un aspecto vital en estos últimos. Esta comunicación intercelular es mediada por moléculas de señalización extracelular y mediadores solubles de diversa naturaleza ^1^, entre los cuales los más estudiados y caracterizados han sido los factores de crecimiento ^2^, las citocinas ^3^ y los neurotransmisores ^4^.

Además de estos, existen las vesículas extracelulares, estructuras con bicapa lipídica secretadas al medio extracelular por múltiples tipos de células. Las diferentes clases de vesículas extracelulares integran redes de comunicación intercelular al transportar un subconjunto específico de proteínas, ácidos nucleicos como el ARNm y el microARN (miARN), y lípidos, todos específicos para las condiciones fisiológicas o patológicas de su célula de origen y que, al parecer, son cargados a las vesículas extracelulares de una forma determinada en lugar de constituir componentes aleatorios ^5^. Así, las vesículas extracelulares permiten el intercambio de información genética a distancia mediante el transporte de ácidos nucleicos, particularmente los miARN, que sobresalen entre las diferentes estructuras y estrategias que tienen las células para comunicarse ^6^.

El interés por el estudio de estas vesículas ha aumentado en la última década, como lo evidencia el auge de publicaciones que estudian su biogénesis, contenido y efectos biológicos, empezando por el estudio de los exosomas y luego ampliándose a otras vesículas extracelulares ^7^^,^^8^, así como la creación de diversas sociedades y recursos. El estudio de los exosomas y otras vesículas extracelulares encierra desafíos analíticos y limitaciones técnicas debido a las características de las muestras. Aunque su desarrollo es vertiginoso, este es un campo científico relativamente joven, por lo que, al plantear proyectos de investigación y estudios de vesículas extracelulares, debe considerarse primero cuál es el estado del campo científico a nivel mundial en cuanto a su nomenclatura y clasificación, así como a las técnicas, recursos, requisitos y especificaciones de calidad, y las instituciones que regulan el campo.

Para alguien nuevo en este campo responder a esta pregunta puede ser un desafío, ya que la información disponible se encuentra dispersa y se centra en la generación de vesículas y el estudio de los efectos prometedores que se les atribuyen, sobre lo cual hay numerosas revisiones y una nomenclatura variada y, en algunos casos, imprecisa ^9^^,^^10^. Son menos los artículos originales que abordan aspectos experimentales y técnicos que las revisiones disponibles ^11^^-^^13^, y son contadas las publicaciones que presentan los requisitos mínimos para los estudios ^7^^,^^14^ y las consideraciones experimentales ^15^^,^^16^. Se encuentran diversas revisiones científicas sobre la situación actual de las técnicas y tecnologías empleadas y sus limitaciones, con recomendaciones de trabajo, pero con información fraccionada.

Por otro lado, se han creado instituciones, bases de datos, recursos bioinformáticos y directrices de trabajo producto del consenso de la comunidad científica, los cuales son usualmente ignorados o se desconocen, por lo que se incurre en errores metodológicos y se ignoran los términos y la nomenclatura oficial, lo que lleva a nombrar la población de vesículas extracelulares obtenida a partir de conceptos erróneos, o cuyo origen e identidad no se ha demostrado adecuadamente. Ello genera falta de homogeneidad y mucha confusión en los resultados reportados y conduce a la imposibilidad de reproducirlos. En este sentido, las instituciones oficiales recomiendan términos y una nomenclatura, pero, hasta donde se pudo comprobar, no hay una publicación que recopile los términos oficiales indexados en la ontologia génica y otros vocabularios controlados. En consecuencia, no siempre se consideran todos los aspectos y recursos de este campo a la hora de plantear proyectos y estudios que resulten integros.

Para solventar este problema, en esta actualización se presenta: primero, la situación actual, introduciendo los términos y la nomenclatura oficial definida para las vesículas extracelulares, analizando su evolución y oficialización, y haciendo recomendaciones sobre su uso; en segundo lugar, compilando la información disponible sobre clasificación de las vesículas extracelulares, técnicas disponibles para su estudio, y las instituciones, guias, bases de datos y recursos bioinformáticos que se han creado en este campo, así como proponiendo perspectivas de avance; se sintetizan, además, los requisitos mínimos y las recomendaciones propuestas a nivel mundial para el desarrollo de estudios sobre estas vesículas, exponiendo y analizando algunos de los desafíos y principales limitaciones asociadas con su estudio y correcta denominación, y se presentan los criterios para el desarrollo de estudios; y, en tercer lugar, se presenta una breve actualización sobre los estudios en instituciones colombianas, contemplando sus avances y fortalezas, analizando de forma crítica la capacidad nacional y las técnicas y tecnologías analíticas disponibles, y resaltando el interés por los estudios de vesículas extracelulares en el país ^17^^-^^29^.

Esta actualización pretende llamar la atención de la comunidad científica interesada en las vesículas extracelulares, y ser una guía para el planteamiento de proyectos y estudios en esta área, presentando las directrices internacionales y las técnicas, así como las limitaciones, retos y oportunidades de su estudio.

Para aquellos investigadores que se acercan por primera vez al campo de las vesículas extracelulares, o que quieran tener un panorama íntegro del área, esta es una guía que los introduce en la nomenclatura oficial, los requisitos mínimos y los criterios necesarios para el desarrollo de estudios, y presenta las técnicas disponibles y más usadas, así como los recursos desarrollados, incluidos los últimos avances científico-técnicos. Darle la debida atención a estos aspectos y al uso correcto de los términos, permitirá plantear proyectos de investigación con calidad internacional que respondan a las exigencias y recomendaciones institucionales.

Por último, el escrito está dirigido, no solo a los investigadores que trabajen con vesículas extracelulares, sino también a revisores, jurados y editores de trabajos en el área como forma de orientación general de la comunidad científica interesada en el campo de estudio de las vesículas extracelulares.

## Definiciones, nomenclatura y clasificación de las vesículas extracelulares

La expresión "vesículas extracelulares" engloba un conjunto heterogéneo de partículas liberadas de forma natural por las células; están delimitadas por una bicapa lipídica y no pueden replicarse, es decir, carecen de un núcleo funcional. Según los registros en PubMed, este término fue usado por primera vez en 1971 por Aaronson, *et al.,* al estudiar su producción en un alga ^30^. La *International Society for Extracellular Vesicles* (ISEV) respalda el uso de este nombre como el término genérico y preferente para denominar estas partículas ^14^. Sin embargo, en los primeros años de estudio fueron múltiples los términos empleados para nombrar las poblaciones de estas vesículas, los cuales se asociaban con su origen tisular y con su función, por ejemplo, prostasomas ^31^, oncosomas ^32^, vesículas de matriz calcificantes ^33^, entre muchos otros, y los genéricos y ampliamente usados exosomas, microvesículas y ectosomas ^34^.

El término "exosoma" fue inicialmente empleado en 1981 por Trams, *et al.,* para nombrar las vesículas extracelulares con actividad enzimática y posible función fisiológica asociada ^35^, y su biogénesis fue descrita por Harding, *et al.*^36^, y por Pan, *et al.*^37^, al estudiar la maduración de los reticulocitos. No obstante, el rol fisiológico de estas vesículas en la presentación de antígenos solo se hizo evidente después de que Raposo, *et al.,* reportaran la liberación de exosomas por parte de linfocitos B ^38^, y Zitvogel, *et al.,* por parte de las células dendríticas ^39^. A partir de ese momento, el estudio de los exosomas suscitó un creciente interés reflejado en el aumento exponencial de publicaciones científicas, especialmente desde el 2004, como lo hicieron patente Lötvall, *et al.,* al comparar la evolución y el uso de los distintos términos en la literatura ^7^. Así, "exosoma" llegó a ser un término ampliamente usado en la literatura biomédica, aunque con diferentes interpretaciones, lo que condujo a un uso impreciso debido a la falta de oficialización y caracterización apropiadas ^8^.

**Figura 1:**
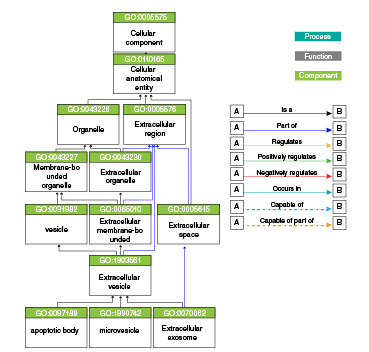
Gráfico de ontología genética para "vesícula extracelular" y algunos términos derivados

Hubo un auge de artículos científicos, en los cuales se usó el término "exosomas" y, posteriormente, el de "microvesículas". Sin embargo, a partir de la fundación de la ISEV en el 2011, estos términos han sido reemplazados progresivamente por el de "vesículas extracelulares", un nombre genérico adoptado por consenso en la comunidad científica internacional asociada en la ISEV y cuyo empleo se generaliza cada vez más ^7^. Este consenso se tradujo en la posterior publicación de los requisitos experimentales mínimos en el documento *Minimal Information for Studies of Extracellular Vesicles* (MISEV) en el 2014 ^7^ y su actualización en el 2018 ^14^, lo cual permitió normalizar y homogenizar la nomenclatura de las vesículas extracelulares y sus diversos tipos, así como su estudio. Lo que define y autoriza un término es su introducción en los vocabularios controlados. Desde el 2014, se contemplan las vesículas extracelulares como parte de la ontología génica, con términos derivados, como los de cuerpos apoptóticos, vesículas extracelulares bacterianas, vesículas de membrana externa bacteriana, exosomas extracelulares, microvesículas, y prominosoma, siguiendo la estructura que se presenta en la figura 1 y las definiciones y sinónimos del cuadro 1 ^40^^-^^42^ (Gene Ontology Project, versión 2021-05-01).

QuickGO - https://www.ebi.ac.uk/QuickGO Extraído de la base de conocimiento de ontología genética, versión 2021-05-01 (43.987 términos de ontología genética | 8.006.434 anotaciones 1.574.201 productos genéticos | 4990 especies) ^40^^-^^42^

**Cuadro 1:** Comparación de los términos indexados en algunos vocabularios o diccionarios controlados

VC	Término	Definición	Año de indexación
GO	*Extracellular vesicles* Z Vesículas extracelulares	Cualquier vesícula que es parte de la región extracelular Sinónimo: micropartícula	2014
	*Apoptotic body* Z Cuerpo apoptótico	Una vesícula que contiene partes de una célula moribunda Los cuerpos apoptóticos pueden variar en tamaño, de 0,8 a 5 um (definición actualizada en el 2016). Sinónimos: vesícula apoptótica, ampolla apoptótica	2011
	*Extracellular exosome* Z Exosoma extracelular	Una vesícula que se libera en la región extracelular por fusión de la membrana endosómica limitante de un cuerpo multivesicular con la membrana plasmática. Los exosomas extracelulares, también llamados simplemente exosomas, tienen un diámetro de aproximadamente 40-100 nm (definición actualizada en el 2016). Sinónimos: exosoma, exosoma vesicular extracelular	2015
	*Microvesicle* Z Microvesícula	Una vesícula extracelular liberada de la membrana plasmática y que varía en tamaño de aproximadamente 100 nm a 1.000 nm (definida en el 2015). Sinónimos: ectosoma, microvesícula extracelular, vesícula de desprendimiento o *shedding*	2015
MeSH - DeCS	*Extracellular vesicles* Z Vesículas extracelulares	Estructuras limitadas por membrana derivadas de membranas celulares y del material citoplasmático, y liberadas al espacio extracelular. Circulan en el fluido extracelular y con la sangre periférica en la microvasculatura donde las células, mucho más grandes, no pueden circular, lo cual afecta una variedad de procesos de comunicación intercelular.	2016
	*Exosomes* Z Exosomas	Tipo de vesícula extracelular que contiene ARN y proteínas, y se secreta en el espacio extracelular por exocitosis cuando los cuerpos multivesiculares se fusionan con la membrana plasmática	2009
	*Cell-derived microparticles* Z Micropartículas derivadas de células	Vesículas extracelulares generadas por el derramamiento de ampollas (sic) de membrana celular	2009

VC: vocabulario controlado; GO: *Gene ontology;* MeSH: *Medical Subject Headings;* DeCS: Descriptores en Ciencias de la Salud Información tomada de 40,41,43,44 y (Gene Ontology Project, versión 2021-05-01)

En 2016, el nombre de vesículas extracelulares fue incorporado en diversos vocabularios terminológicos controlados, entre ellos, el de *Extracellular Vesicles* en el *Medical Subject Headings* (MeSH) de la *U.S. National Library of Medicine* - NLM ^43^, y, además, "vesículas extracelulares" se incluyó en los Descriptores en Ciencias de la Salud (DeCS) de la Biblioteca Virtual en Salud (BVS) de la Organización Panamericana de la Salud ^44^. Esta indexación tardía, cinco años después de la fundación de la ISEV y dos años después de la publicación del MISEV 2014, y la inclusión del término en la ontología génica, ocurrió bastante tiempo después del establecimiento de sus términos derivados, incluidos desde el 2009 en los DeCS y los MeSH como "Exosomas" / "Exosomes" y "Micropartículas Derivadas de Células" / "Cell-Derived Microparticles" (cuadro 1). Por otro lado, el término "ectosoma", empleado en los MISEV 2018 para agrupar a las micropartículas y microvesículas ^14^, y definido en diversos artículos recientes ^5^^,^^45^, no ha sido incorporado a los DeCS y aparece como una entrada bajo el término "Cell-Derived Microparticles" en los MeSH, por lo cual su empleo es dispar.

Esta indexación tardía y espaciada pudo repercutir en una falta de homogenización y precisión de los términos usados en los artículos publicados hasta ese momento, en los que se empleaba el término "exosoma" de forma general, y en algunos casos sin demostrar su origen endosomal ni sus características específicas, contrario a la posición adoptada por la ISEV y sus recomendaciones.

Además, en un esfuerzo mancomunado, varios investigadores del área actualizaron la ontología génica con énfasis en el ARN extracelular (exARN), lo que se refleja actualmente en esta base de conocimiento y en su clasificación de los términos asociados con las vesículas extracelulares y los exARN ^46^. En ese documento, Cheung, et *al.,* proponen que cualquier término futuro agregado a la ontología génica, que represente los tipos de vesículas que forman parte de la región extracelular, se clasifique automáticamente como 1903561 "vesícula extracelular" Sin embargo, los tamaños propuestos por los autores para definir cada tipo de estas vesículas difieren de los establecidos actualmente en la ontología génica (cuerpo apoptótico =50-5000 nm, exosoma =40-200 nm, microvesícula =50-1000 nm), lo que demuestra la falta de consenso que todavía existe en cuanto al tamaño de cada subtipo de vesículas extracelulares (cuadros 1 y 2).

**Cuadro 2:** Comparación de las características de algunos tipos de vesículas extracelulares

Características	Exosomas	Ectosomas	Microvesículas	Cuerpos apoptóticos
Mecanismo de generación	Exocitosis de MVB	Brote de la membrana plasmática	Brote de la membrana plasmática	Liberación de vesículas de células apoptóticas
Tamaño	40 - 100 nm ^a^ 50 - 100 nm ^b^	100 - 200 / 500 nm, según publicación ^b^	100 - 1000 nm ^a b^	0,8 - 5 μm ^a^ 1 - 5 μm / 50 -500 nm ^b^
Composición lipídica	Enriquecidas en colesterol, esfingomielina y ceramida; contienen balsas lipídicas; exponen fosfatidilserina ceramida grande.	Enriquecidas en colesterol y diacilglicerol; exponen fosfatidilserina y ceramida.	Exponen fosfatidilserina.	ND
Marcadores proteicos	Tetraspaninas (CD81, CD63, CD9, CD61), Alix, LAMP1 y Tsg101; unión a anexina V	*CR1, TyA, C1q, enzimas proteolíticas. No CD63.*	Integrinas, selectinas y ligando CD40; unión a anexina V, factores tisulares y marcadores específicos celulares	Unión a anexina V, contenido de ADN

ND: no determinado

^a^ definida en GO

^b^ definida según la literatura

Basado en: ^45^^,^^47^^,^^48^ (Gene Ontology project, versión 2021-05-01)

Con base en el conocimiento actual, las vesículas extracelulares generadas por células eucariotas se pueden clasificar en: exosomas, microvesículas/ectosomas y cuerpos apoptóticos, según su origen o biogénesis. Los exosomas son definidos por su origen endosomal, y los ectosomas y microvesículas como derivados de la membrana plasmática. en tanto que los cuerpos apoptóticos se forman de células apoptóticas. Asimismo, las vesículas extracelulares se pueden describir por su tamaño y composición molecular. En diversas publicaciones se comparan estos tipos de vesículas en función de su tamaño, sedimentación, densidad en sacarosa, apariencia por microscopía electrónica, composición química, y mecanismo de generación, entre otras características ^45^^,^^47^^,^^48^. Sin embargo, aún no se ha llegado a un consenso en cuanto a marcadores específicos de cada tipo de vesícula extracelular, ya que asignar un tipo a una ruta de biogénesis en particular es difícil, a menos que se detecten en el momento de su liberación mediante técnicas de observación celular en vivo; no obstante, se cuenta con diversas moléculas que permiten describir cada subtipo diferenciando entre ectosomas y microvesículas (cuadro 2).

A diferencia del estado inicial, actualmente se cuenta con una nomenclatura definida en las bases de conocimiento y en los vocabularios controlados, lo que permite la homogenización y el uso preciso de los términos por parte de los investigadores del área. Sin embargo, aún se presentan algunas inconsistencias, pues no se adoptan uniforme y rápidamente dichos términos controlados. Por ejemplo, algunos investigadores usan el término "microvesículas" para referirse a las vesículas extracelulares grandes (>200 nm) y el de "exosoma" para las pequeñas (≈30 nm a 200 nm), lo cual es incorrecto, ya que desconoce su origen y definición biogénica. Esta definición basada en el tamaño, aunque práctica, es mecánicamente estéril, y no ha sido aprobada por las autoridades científicas. Así, se debe promover el uso correcto de los términos oficiales en los documentos y estudios del área, concordante con las técnicas y metodologías empleadas, como se ilustra más adelante. A medida que evolucione el campo y aumente el conocimiento científico, se esperan marcadores y características adicionales que permitan diferenciar y definir de forma más precisa términos actualmente sinónimos, como microvesículas y ectosomas, entre otros.

## Organización y evolución del campo a nivel internacional, creación de instituciones, criterios para desarrollar estudios y desarrollo de recursos

El auge de los estudios en el campo de los exosomas y otras vesículas extracelulares, y la falta de un consenso y rigor que permitiera su reproducibilidad, motivaron la creación de un conjunto de sociedades nacionales, y de una sociedad internacional que ha liderado la creación de recursos, espacios de formación y divulgación e iniciativas de estandarización. A nivel internacional, existe la *International Society for Extracellular Vesicles* (ISEV) (https://www.isev.org/) fundada en el 2011 por investigadores interesados en su estudio, incluidos el de los exosomas y microvesículas. Nació a raíz del *International Workshop on Exosomes* y, en el 2012, celebró su primera reunión y realizó el lanzamiento de la revista oficial de la Sociedad: *Journal of Extracellular Vesicles* (JEV) ^49^.

En ese mismo año, se estableció la *American Society for Exosomes and Microvesicles* (ASEMV) (https://www.asemv.org/), la cual colabora con otras organizaciones científicas y con los *National Institutes of Health* (NIH) de Estados Unidos en la detección de oportunidades en el campo, al tiempo que patrocina conferencias científicas; su ámbito incluye también los estudios sobre las vesículas extracelulares, los exARN y las vesículas de membrana externa bacteriana. La revista oficial de la ASEMV, la *Exosomes and Microvesicles* (EXMV), fue lanzada en el 2013; esta se orienta a la aplicación de exosomas y microvesículas en el mantenimiento de la salud humana, y el desarrollo de estrategias para diagnosticar y combatir mejor las enfermedades ^50^.

Asimismo, se han creado diversas sociedades nacionales y grupos en Europa: la *UK Society for Extracellular Vesicles* (UKEV) (https://www.ukev.org.uk/) a partir del Foro UKEV realizado en el 2014; la *German Society of Extracellular Vesicles* (GSEV) (https://www.extracellular-vesicles.de/), creada en el 2017; el grupo español de innovación e investigación en vesículas extracelulares (GEIVEX) (https://www.geivex.org/) en el 2012; la *Italian Society for Extracellular Vesicles* (EVIta), la *Austrian Society for Extracellular Vesicles* (ASEV), la *Norwegian Regional Research Network on Extracellular Vesicles* (RRNEV) y la *French Society of Extracellular Vesicles* (FSEV). En el resto del mundo, se han creado la *Japanese Society of Extracellular Vesicles* (JSEV), la *Society for Clinical Research and Translation of Extracellular Vesicles Singapore* (SOCRATES) y la *Brazilian Society for Extracellular Vesicles* (BSEV), entre otras.

Ante la necesidad de establecer una nomenclatura con términos y definiciones precisas, así como criterios para caracterizar las vesículas extracelulares apropiadamente, la ISEV ha organizado seminarios y talleres que reúnen a expertos científicos e investigadores en el campo para discutir cómo abordar las lagunas existentes en el conocimiento y las técnicas disponibles para afrontar tales vacíos. Los talleres iniciales sirvieron como antesala a la publicación de los primeros artículos de posición de la ISEV y de las guías MISEV. En el taller del "Seminario de Investigación ISEV: Análisis y función del ARN en VE (evRNA)" del 2012, sesionaron mesas de discusión en las que se proporcionó un marco basado en la evidencia para el aislamiento y análisis de vesículas extracelulares, la purificación y el análisis de moléculas de ARN asociadas, y la ingeniería molecular de las vesículas extracelularespara intervenciones terapéuticas ^51^. Las conclusiones de estas mesas se publicaron como artículos de posición en el 2013, uno sobre tecnologías de análisis de evRNA y su análisis bioinformático ^52^, y otro en que se abordaba la estandarización de la recolección de muestras, el aislamiento y los análisis de vesículas extracelulares ^13^. Este último incluye una revisión de los materiales publicados y la experiencia de los autores, y hace énfasis en la necesidad de estandarizar el manejo de muestras, los controles normativos apropiados y la evaluación de las técnicas de aislamiento y análisis para facilitar la comparación de resultados.

Producto de estas discusiones, en el 2014, la ISEV publicó los requisitos experimentales mínimos de estudios, MISEV 2014, donde se establecieron estándares para el estudio de las vesículas extracelulares, principalmente los exosomas, presentando técnicas para caracterizar la muestra y estableciendo la necesidad de marcadores apropiados que permitan declarar la presencia de dichas vesículas en los aislamientos, así como recomendaciones sobre los controles adecuados para los estudios de su actividad funcional ^7^.

Para ofrecer una guía más detallada, que reflejara un mayor consenso de la comunidad científica y se nutriera de la experiencia de otros autores, en el 2015 se llevó a cabo la primera gran encuesta detallada de las prácticas mundiales usadas hasta el momento para el aislamiento y la caracterización de las vesículas extracelulares ^10^, cuyos resultados influyeron en la posición oficial de la ISEV y llevaron a la actualización de las guías MISEV publicada en noviembre del 2018 ^14^. Además de facilitar la reproducibilidad de los ensayos y análisis y avanzar en la estandarización de las técnicas empleadas, en esta nueva guía se consideran los avances y experiencias del campo y se actualizan las recomendaciones basadas en la evidencia científica disponible en el momento.

Cabe resaltar que la guía MISEV 2018 no propone marcadores moleculares para caracterizar específicamente cada subtipo de vesícula extracelular, desestimula el uso del término "exosoma" eliminando las recomendaciones para caracterizar exosomas y las proteínas que se espera encontrar en estos, y recomienda el empleo de "vesículas extracelulares" como nombre genérico, a diferencia de la guía MISEV 2014, en la cual algunas sugerencias estaban sesgadas por una visión de las vesículas extracelulares "orientada a los exosomas" Este cambio de criterios y recomendaciones refleja una evolución en la comprensión de los subtipos de vesículas extracelulares y sus asociaciones con otras entidades, al tiempo que busca aumentar el rigor y la reproducibilidad de los estudios que las involucran.

Para responder a la necesidad de transparencia y rigor, incrementar la reproducibilidad y facilitar la estandarización de la investigación sobre vesículas extracelulares, se estableció un consorcio internacional que, en el 2017, desarrolló la base de conocimiento EV-TRACK (http://evtrack.org/), repositorio en línea que centraliza los datos con base en las características de las vesículas extracelulares y los métodos empleados siguiendo las recomendaciones de las guías MISEV, e incluye una herramienta de búsqueda de artículos científicos y de material sin publicar destinada a la consulta de revisores y editores.

La plataforma EV-TRACK tiene siete características que ayudan a los investigadores a:

1. subir la información de sus experimentos,
2. asignar la métrica EV-METRIC a cada experimento,
3. consultar la base de datos de artículos,
4. entrenarse y familiarizarse con los parámetros experimentales más relevantes de las guías MISEV,
5. buscar nuevos métodos al permitir las anotaciones de la comunidad,
6. catalogar sistemáticamente las características bioquímicas y físicas de las vesículas extracelulares que dan información sobre su biología, y
7. incluir a la comunidad como consorcio y permitir la participación de los usuarios en las decisiones futuras ^53^.

Cuenta, además, con la EV-METRIC, una métrica diseñada para representar el grado de transparencia en el reporte de parámetros experimentales, la cual está habilitada para el envío de datos previos al sometimiento de un manuscrito y como herramienta de búsqueda de artículos publicados. La base de datos de artículos se puede consultar según las características y los parámetros de los estudios, como se desglosa en el cuadro 3; los parámetros EV-METRIC son una lista de chequeo. La consulta del material publicado permite realizar metaanálisis de experimentos y seguir la situación actual del tema. Además, la herramienta destinada a revisores y editores incluye una lista de chequeo orientada a garantizar la transparencia de los aspectos metodológicos de los experimentos, la cual consta de 115 parámetros para catalogar características específicas de los métodos de aislamiento y caracterización de las vesículas extracelulares, presentada en su tabla suplementaria 1 ^53^.

**Cuadro 3:** Opciones de consulta y parámetros incluidos en EV-TRACK

Opciones de consulta habilitadas en EV-TRACK	
Especie	múltiples: 12 mamíferas y 59 no mamíferas
Tipo de biofluido	sobrenadante de cultivo celular, plasma sanguíneo, suero, orina, leche, semen, saliva, ascitis, fluido cerebroespinal, fluido de lavado broncoalveolar
Objetivo del estudio	biogénesis/clasificación, biomarcador, función, ómica y técnica
Método de separación	comercial, cojín de densidad (density cushion), gradiente de densidad, ultracentrifugación diferencial, filtración, inmunoafinidad, cromatografía de exclusión de tamaño, ultrafiltración, microfluidos
Método de análisis proteico	ELISA, citometría de flujo, microscopía inmunoelectrónica, proteómica, western blot
Método de análisis de partículas	fraccionamiento en flujo mediante campo de flujo asimétrico, microscopía de fuerza atómica, dispersión de luz dinámica, microscopía electrónica, citometría de flujo de alta resolución, análisis de rastreo de nanopartículas, detección de pulso resistivo sintonizable
Parámetros EV-METRIC:	proteína enriquecida en VE proteína no enriquecida en VE análisis cualitativo y cuantitativo
	imágenes de microscopía electrónica gradiente de densidad densidad de VE
	especificaciones de ultracentrifugación especificaciones de anticuerpos preparación del lisado
Parámetros experimentales relacionados con la caracterización de VE	
Análisis de proteínas	Proteínas enriquecidas en VE: análisis de tres o más proteínas enriquecidas en VE
	Proteína no enriquecida en VE: evaluación, al menos, de una proteína no enriquecida en VE
	Características específicas del anticuerpo: clon del anticuerpo / número de referencia y dilución ^*^
	Preparación de lisado: composición de la solución tampón de lisis o descomposición usada, o número de referencia para soluciones tampón de descomposición usadas *
Análisis de partículas	Análisis cualitativo y cuantitativo: implementación de métodos cualitativos (por ejemplo: EM, AFM) y cuantitativos (por ejemplo: NTA, DLS, TRPS, citometría de flujo de alta resolución). Para el método cuantitativo, se espera informar la concentración de partículas.
	Imágenes de microscopía electrónica: inclusión de una imagen de microscopía electrónica de campo amplio y de primer plano
Parámetros experimentales relacionados con la separación de VE	
Gradiente de densidad: gradiente de densidad realizado al menos como validación si los resultados descritos se atribuyen a las VE	
Densidad de VE: reporte de la densidad obtenida de las VE	
Características específicas de ultracentrifugación: informe de RCF o fuerzas g, duración de los pasos de ultracentrifugación y tipo de rotor +	

* Pueden considerarse "no aplicables", dependiendo de los métodos de caracterización implementados en un estudio en particular.

+ Pueden considerarse "no aplicables", dependiendo del método de separación implementado en un estudio particular. Información disponible en: http://evtrack.org/

La plataforma EV-TRACK es una gran herramienta para investigadores del área de las vesículas extracelulares y de otros campos, ya que las anotaciones de su base de datos, las opciones de consulta y la información presentada, facilitan la interpretación y replicación de experimentos publicados en artículos, y centraliza la información sobre la biología y la metodología de las dichas vesículas. Con esta herramienta se puede filtrar por el tipo de aislamiento y caracterización de las vesículas extracelulares y los parámetros EV-METRIC reportados, y obtener las propiedades biofísicas y moleculares de las vesículas extracelulares. Esto permite que los usuarios evalúen la calidad de la preparación de la muestra de vesículas extracelulares y los datos correspondientes obtenidos; sin embargo, un valor elevado de EV-METRIC solo implica que los datos publicados están bien anotados.

Estos parámetros fueron acordados por el consorcio de EV-TRACK como indispensables para una interpretación inequívoca que permita una reproducción independiente de los experimentos. Aun cuando los investigadores desconozcan o no sigan las guías MISEV, EV-TRACK pretende entrenarlos en el uso de la métrica EV-METRIC y estimular la puesta en práctica de las guías MISEV. Este recurso está en continuo desarrollo y los parámetros incluidos pueden ser modificados, aumentados o refinados con el avance del campo. Por ejemplo, en el caso del análisis de partículas, deben considerarse la pureza y la calidad del vehículo empleado para suspender las vesículas extracelulares, y usarlo como control de partículas durante los análisis, específicamente para los análisis de rastreo de nanopartículas, así como el medio de cultivo para el caso de sobrenadantes de cultivos celulares. No obstante, la clave de su éxito reside en su implementación generalizada por parte de la comunidad científica.

Por otro lado, y gracias a la gran cantidad de resultados obtenidos en el área, se han desarrollado diversas bases de datos de las moléculas identificadas en las vesículas extracelulares, entre ellas, EVpedia Exocarta, Vesiclepedia, y EVmiRNA. Creadas a modo de repositorios antes de la creación de EV-TRACK, están basadas en la web y son de acceso libre. EVpedia condensa datos provenientes de experimentos de alto rendimiento, como análisis proteómicos de vesículas extracelulares derivadas de células procariotas y eucariotas ^54^^,^^55^ (http://evpedia.info/); infortunadamente, está inactiva actualmente.

El laboratorio del profesor Mathivanan desarrolló ExoCarta y Vesiclepedia, las cuales compilan el ARN, las proteínas, los lípidos y otros metabolitos, identificados en exosomas de múltiples organismos ^56^^,^^57^ (http://www.exocarta.org/) y en diversos tipos de vesículas extracelulares ^58^^,^^59^ (http://microvesicles.org/), respectivamente. Ambas bases tienen curaduría manual y se alimentan de estudios publicados y sin publicar o en prensa, principalmente estudios proteómicos y lipidómicos, que permiten buscar y descargar el contenido sobre vesículas extracelulares con base en criterios de búsqueda, como nombre del gen, miARN, organismo, tipo de contenido y de muestra, y, en el caso de Vesiclepedia, tipo de vesícula. Asimismo, las dos bases de datos muestran las técnicas empleadas en cada estudio e hipervínculos al registro en PubMed. Exocarta incluye una lista de verificación del cumplimiento de los estándares definidos por la ISEV y, Vesiclepedia, el código y el vínculo para el registro en EV-TRACK y el porcentaje de EV-METRIC. Sin embargo, estas bases de datos presentan algunos vínculos rotos y, como toda base de datos, requieren de actualización permanente.

Se han creado, además, recursos enfocados en el ARN extracelular (exARN), libre o asociado con las vesículas extracelulares. El *Extracellular RNA Communication Consortium* (ERCC) se creó como una iniciativa de las sociedades ASEMV e ISEV, y agrupa a los investigadores para abordar los problemas críticos en el campo de investigación del exARN. Es financiado como una iniciativa del fondo común de los NIH, y cuenta con un portal de investigación de exARN donde se dispone de recursos, proyectos y publicaciones (https://exrna.org/) ^60^; además, incluye la base de datos exRNA Atlas, un repositorio de perfiles de exARN derivados de la secuenciación de ARN pequeños y qPCR de biofluidos humanos y de ratón, que permite seleccionar, ver y descargar los datos y visualiza el cumplimiento de los estándares de calidad de datos del ERCC (https://exrna-atlas.org/) ^61^. Asimismo, se cuenta con la base de datos EVmiRNA, en la cual se reúne el perfil de los miARN obtenidos de las vesículas extracelulares (http://bioinfo.life.hust.edu.cn/EVmiRNA) ^62^.

Además, en el 2015, se desarrolló la herramienta bioinformática FunRich, que permite hacer análisis de enriquecimiento funcional del set de datos sobre vesículas extracelulares y generar múltiples representaciones gráficas de ellos. Es una herramienta de acceso abierto para analizar el conjunto de datos 'ómicos' de múltiples tipos y organismos (http://www.funrich.org/) ^63^. Esta herramienta fue mejorada con base en los aportes y sugerencias de la comunidad científica y, actualmente, está integrada como complemento de Exocarta y Vesiclepedia para ayudar al usuario a analizar por bioinformática los datos depositados en dichas bases. La versión actual (3.1.3) permite realizar enriquecimientos de miARN y descargar información automáticamente de las bases de datos asociadas ^64^; y se encuentra en constante desarrollo gracias a que involucra a los usuarios en el desarrollo del *software,* propiciando un trabajo conjunto, favoreciendo el desarrollo de una herramienta de análisis integral y respondiendo de esta forma a las necesidades de la comunidad científica.

En cuanto a los recursos educativos, la ISEV lanzó en el 2016 su primer curso en línea para estudiantes y principiantes en el campo de las vesículas extracelulares ^65^. Se trata de un curso masivo ofrecido de forma gratuita bajo el nombre de "Basics of Extracellular Vesicles" ofrecido por la Universidad de California en Irvine a través de la plataforma Coursera (https://es.coursera.org/learn/extracellular-vesicles). En Europa, existe la red TRAIN-EV *(Training in Extracellular Vesicles),* que reúne a un grupo de sociedades y científicos europeos, coordina proyectos multidisciplinarios e intersectoriales para crear sinergias entre las actividades de investigación sobre vesículas extracelulares, desarrolla un programa de entrenamiento en investigación cuyo objetivo es proporcionar una capacitación excelente e integrada a jóvenes investigadores *(Early Stage Researchers)* y, simultáneamente, adelanta investigaciones innovadoras para abordar las brechas del campo y generar nuevo conocimiento. La TRAIN-EV se creó en el 2017 como un megaproyecto financiado por el programa Horizonte 2020 de la Unión Europea y, hasta el momento, ha vinculado a 16 jóvenes investigadores que desarrollan proyectos que abordan desde aspectos técnicos, comparación de plataformas, desarrollo de dispositivos, materiales de referencia biológica y herramientas bioinformáticas, hasta caracterización y aspectos funcionales y de relevancia biológica (http://train-ev.eu/). Ello demuestra el desarrollo de este campo científico y el interés de sus miembros en superar los retos y desafíos experimentales inherentes a él.

## Retos y requisitos mínimos para el desarrollo de estudios

Los llamados "exosomas" son las vesículas extracelulares que han recibido mayor interés. Sin embargo, usualmente son difíciles de reconocer ya que, una vez secretadas, se confunden con las otras vesículas secretadas por rutas diferentes, siendo compleja su identificación tal como son definidos: por su origen celular o ruta de biogénesis. Esto se debe a que sus propiedades biofísicas (cuadro 2), los cuales suelen estar presentes en las preparaciones que pretenden aislar únicamente exosomas ^66^. Por lo tanto, es vital considerar las condiciones experimentales preanalíticas y de aislamiento de las vesículas extracelulares, y caracterizarlas adecuadamente.

A pesar del origen intracelular definido para cada población de vesículas extracelulares descrita, aún no hay marcadores específicos de cada una de estas poblaciones que permita distinguir entre ellas una vez secretadas. Se ha visto que diversos marcadores que inicialmente se consideraban como específicos para exosomas, también se presentan en vesículas extracelulares de otros orígenes.

En un estudio en el que se inhibió la Rab27a, una GTPasa pequeña necesaria para la secreción de exosomas, hubo un descenso en la secreción vesicular de algunos marcadores convencionales de exosomas, incluidas la tetraspanina CD63, las proteínas implicadas en la formación de endosomas multivesiculares Tsg101 y Alix, y la proteína de choque térmico Hsc70, pero no así de otros marcadores como la tetraspanina CD9 y la proteína asociada con la membrana periférica Mfge8. La CD9 y la Mfge8 se encontraron en otras vesículas extracelulares diferentes a exosomas y se vieron, al menos, dos poblaciones distintivas de vesículas extracelulares después del aislamiento por ultracentrifugación diferencial ^67^.

Este trabajo reveló la heterogeneidad de las preparaciones de vesículas comúnmente conocidas como exosomas, y que el protocolo de ultracentifugación diferencial empleado hasta el momento para aislarlos ^68^ purifica concomitantemente vesículas de otros orígenes, por lo que la fracción de vesículas extracelulares obtenida a 100.000g corresponde, en realidad, a vesículas extracelulares pequeñas *(small EV,* sEV), incluidos los exosomas, por lo que se debe comprobar su enriquecimiento en estos.

Más recientemente, en un análisis proteómico cuantitativo de poblaciones de vesículas extracelulares obtenidas mediante diversos métodos específicos, se vio que los marcadores que se pensaba que eran exclusivos de los exosomas, como las moléculas del complejo mayor de histocompatibilidad, MHC, de clases I y II, la flotilina, la actina, la Hsc70/Hsp73 de expresión constitutiva y la Hsp70/Hsp72 inducible, estaban presentes de forma similar en todos los tipos de vesículas extracelulares y, por lo tanto, no podían considerarse marcadores específicos de exosomas ni de vesículas extracelulares pequeñas (aisladas a 100g); asimismo, se confirmó que la población de vesículas extracelulares pequeñas era una mezcla de subpoblaciones de exosomas y vesículas extracelulares no exosomales y que los marcadores de exosomas anexina XI, V y VI, ADAM10, ACE, EHD4 y flotilina, independientemente de estar asociados o no con una tetraspanina, realmente eran genéricos de las vesículas extracelulares pequeñas; también, se estableció que la tetraspanina CD63, empleada para definir y aislar exosomas de vesículas extracelulares pequeñas, se presentaba en las grandes ^69^. Así, es necesario emplear más de un marcador y considerar las condiciones preanalíticas y analíticas de aislamiento empleadas, pues son las que determinan el tipo de vesículas extracelulares que se va a obtener.

Por otro lado, en los últimos años, se ha desarrollado un gran número de técnicas para el aislamiento y caracterización de vesículas extracelulares. Sin embargo, la estandarización de varias de ellas es escasa, lo que conduce a que los cambios en los protocolos generen conjuntos de vesículas extracelulares diferentes y de calidad variable, prácticas inadecuadas que llevan a afirmaciones incorrectas por el desconocimiento de la técnica y la naturaleza de la matriz y las vesículas. En últimas, el reporte incompleto de las condiciones experimentales empleadas, la desatención de algunas condiciones o la omisión de algunos pasos y análisis, conducen a la falta de reproducibilidad. Para superar estos retos y dificultades, nacen las guías MISEV, ya presentadas anteriormente.

La guía MISEV 2014 proporcionaba recomendaciones sobre los métodos experimentales y la información mínima requerida en la presentación de informes; es un primer paso hacia la homogenización y estandarización en tres áreas clave: aislamiento y purificación, caracterización y estudios funcionales de vesículas extracelulares ^7^. En la guía MISEV 2018 se validan algunas de las primeras recomendaciones y se las amplía gracias al avance en el conocimiento. Asimismo, se introducen nuevas advertencias y aspectos, por ejemplo, la cuantificación durante los pasos de caracterización y el análisis de la topología de los componentes asociados con las vesículas extracelulares; además, se elimina la referencia a "exosomas" y las proteínas esperadas o no en ellos a favor del término genérico de "vesículas extracelulares" definiendo los subtipos con base en sus características físicas y bioquímicas y la fuente o condición de origen ^14^. En el cuadro 4 se presentan las principales recomendaciones y una comparación de estas guías.

**Cuadro 4:** Comparación de las recomendaciones de MISEV 2014 y de MISEV 2018

	Principales recomendaciones de MISEV 2014	Actualización en 2018
Aislamiento / purificación de VE	No existe un único método de aislamiento óptimo, así que elija en función de las aplicaciones posteriores y la pregunta científica.	Alta recuperación, baja especificidad: estuches de precipitación basados en polímeros, filtros de centrífuga con cut-off de bajo peso molecular sin pasos adicionales de separación, y ultracentrifugación de alta velocidad sin pasos adicionales de separación Recuperación intermedia, especificidad intermedia: ultracentrifugación diferencial con tiempos y velocidades intermedios incluyendo o no pasos de lavado, cromatografía de exclusión por tamaño, filtros de centrífuga de alto peso molecular como paso inicial, columnas de afinidad de membrana y filtración de flujo tangencial Baja recuperación, alta especificidad: filtración, gradiente de densidad, afinidad
	Informe todos los detalles de los métodos para permitir la reproducibilidad.	Depositar los detalles experimentales con EV-TRACK. Esta base de conocimientos facilita la presentación de informes sobre métodos.
		Algunos protocolos, incluidos los asociados con estuches comerciales, pueden generar poblaciones de VE unidas o mezcladas con los componentes introducidos. Al realizar experimentos funcionales, incluir controles de procedimiento y aumentar la separación de las VE
Caracterización de VE	Caracterización general. Mostrar:	a) Mínimo tres marcadores proteicos positivos de VE, incluido al menos uno transmembrana /proteína unida a lípidos, y una proteína citosólica b) Al menos un marcador de proteína negativo	Categorías adicionales de proteínas por considerar para la caracterización: a) marcadores proteicos positivos de VE, asociados a la membrana y al citosol b) marcadores proteicos negativos (control de pureza) c) marcadores específicos de un subtipo de VE d) proteínas extracelulares solubles con actividad funcional
	Caracterizacion de vesiculas individuales. Utilice dos técnicas diferentes y complementarias, como:	a) microscopia electronica o de barrido por sonda (mostrar tanto campo amplio como cercano) b) seguimiento de partículas individuales	
			Describir cuantitativamente tanto la fuente como la preparación de VE
			Análisis topológico de los componentes asociados con las VE, al menos para aquellos con una función dada asociada con VE
Actividad biológica asociada a VE	Debería incluir: a) Estudios de dosis-respuesta		Adicional: comparación cuantitativa de la actividad del medio condicionado o biofluido 1) antes, 2) después de la eliminación de las VE, y 3) de las VE en sí, teniendo en cuenta que la fracción de VE puede incluir materiales aislados concomitantemente o contaminantes. Control sugerido adicional: comparación cuantitativa de la actividad de los subtipos de VE deseados contra los "descartados"
		b) Controles negativos o background del proceso para descartar la influencia de los componentes del suero u otros posibles contaminantes		Para los biofluidos, controles negativos de las funciones asociadas con la enfermedad = fluidos de donantes sanos, sin tratar o de otro tipo adecuado
		c) Controles para evaluar la influencia de componentes solubles o macromoleculares no-VE * c-i) Gradientes de densidad u otro método para mostrar que la actividad es intrínseca a la VE, no solo asociada c-ii) Remoción de VE para reducir la actividad c-iii) Marcación de VE o celular (ej.: marcado fluorescente, con interpretación cuidadosa)		Realizar ensayos funcionales después de separaciones rigurosas, comparando las fracciones VE y no VE para identificar qué proporción de actividad está asociada con cada fracción (en caso de que no sean VE)

^*^ Una de estas opciones ^7^^,^^14^

Es necesario insistir en que se debe nombrar adecuadamente cada subtipo de vesículas extracelulares aisladas según las definiciones oficiales, esto es, la ruta de biogénesis que las originó. Sin embargo, rastrear las vesículas aisladas hasta su ruta de biogénesis es difícil en el caso de cultivos celulares y, por ahora, es imposible en el caso de fluidos biológicos obtenidos de sistemas vivos, ya que, una vez son secretadas las vesículas de interés, se mezclan con otros tipos de vesículas que pueden ser aisladas concomitantemente en las preparaciones y, como ya se explicó, no existe una forma inequívoca y general de distinguirlas con base en sus propiedades biofísicas (cuadro 2) o en su contenido molecular únicamente. Esto, sumado a los pocos marcadores para caracterizar y definir adecuadamente cada subtipo vesículas, dificulta la definición exacta de aquellas que se aíslan.

Para superar dicho escollo, en la guía MISEV 2018 se propone que la expresión "vesículas extracelulares" sea usada como una base sobre la cual se añadan progresivamente detalles físicos y moleculares para designar cada población y subtipo, indicando su origen, condiciones de aislamiento, tamaño y los marcadores positivos o negativos a medida que se avanza en su caracterización ^14^. Por ejemplo, la denominación "sEV tumoral 100K CD9+ CD81+ Cyt C-" denota una población de vesículas extracelulares pequeñas, derivada de células tumorales, aislada a 100.000g, positiva para las tetraspaninas CD9 y CD81, y negativa para la proteína mitocondrial citocromo C, lo cual podría incluir exosomas y ectosomas según lo discutido previamente.

Además, las técnicas actuales permiten monitorear el ensamblaje, la producción y la secreción de las vesículas extracelulares y, por lo tanto, definir qué rutas de biogénesis están activas antes de su liberación lo cual permite inferir los tipos que se espera aislar y contribuye a su caracterización.

En este trabajo, no se contempló la revisión comparativa de las técnicas disponibles, ya que la literatura cuenta con diversos artículos y revisiones de este tipo que presentan y analizan técnicas de aislamiento y caracterización, incluidas, en algunos casos, consideraciones, conceptos erróneos comunes, dificultades metodológicas y referencias de su aplicación (16 66,70-73). No obstante, se resumen a continuación las técnicas disponibles, su clasificación y los aspectos clave recomendados en las guías MISEV que deben tenerse en cuenta (cuadro 4), resaltando los últimos avances.

## Consideraciones para la fase preanalítica

Antes del aislamiento y caracterización de las vesículas, es necesario considerar la muestra de origen, las condiciones a las que está sometida y su almacenamiento. En general, las vesículas extracelulares se pueden aislar de medios condicionados por cultivos celulares y de fluidos biológicos, cada uno sujeto a consideraciones específicas y buenas prácticas asociadas. Se ha descrito el aislamiento de vesículas extracelulares, incluido el de exosomas y microvesículas, en fluidos tan variados como sangre, orina, leche materna, saliva, lágrimas, semen, líquido amniótico, líquido ascítico, líquido cefalorraquídeo y bilis, entre otros ^34^.

Por otro lado, las condiciones preanalíticas y de obtención de vesículas extracelulares dependerán de los ensayos y análisis posteriores que se quieran realizar, ya que cada uno presenta limitaciones y requisitos diferentes, y es sensible a diversos tipos de contaminantes. Un análisis proteómico basado en la espectrometría de masas es sensible a todo tipo de contaminantes proteicos, como las proteínas séricas en el caso de la sangre, y de medios condicionados por cultivos suplementados con suero. A diferencia, la identificación de ARNm y miARN específicos mediante qPCR puede ser más tolerante a ácidos nucleicos contaminantes de otras especies (en función de la secuencia), pero no podrá discriminar entre ARN encapsulado o adherido a las vesículas o libre, a no ser que ello se controle en la fase preanalítica y de aislamiento. Por lo tanto, las consideraciones preanalíticas y los contaminantes que deban controlarse dependerán de la matriz en la que se encuentren las muestras, las vesículas extracelulares objeto de estudio y las aplicaciones posteriores.

En diversas revisiones se han centrado en la recolección, almacenamiento y manejo de la muestra, brindando consideraciones adicionales y recomendaciones para un buen trabajo experimental ^13^^,^^16^, tanto en fluidos biológicos, especialmente la sangre, como en medios condicionados por cultivos celulares. Los fluidos biológicos tienen necesariamente un origen celular mixto y las consideraciones se enfocan en la obtención de la muestra. En el caso de la sangre como material de partida, se puede trabajar con suero o plasma, aunque se recomienda este último, en tanto que la escogencia de los anticoagulantes dependerá de los análisis posteriores; la relación entre sangre y anticoagulante debe ser apropiada y es necesario controlar la cantidad de plaquetas residuales, ya que estas contaminan la muestra.

En el caso de los medios condicionados por cultivos celulares, las buenas prácticas incluyen garantizar la calidad del cultivo celular, incluida su identidad, la ausencia de contaminación microbiana, especialmente micoplasma, una baja tasa de muerte celular para evitar contaminación con cuerpos apoptóticos, el número de células que está condicionando el medio y por cuánto tiempo, entre otros. Si el medio de cultivo se suplementa con suero, es vital reconocer que este contiene vesículas extracelulares que van a contaminar la preparación y se deben controlar; de otra forma, los análisis posteriores estarán alterados por las vesículas extracelulares derivadas del suero.

Entre los aspectos cruciales que no siempre se garantizan o reportan en los protocolos y métodos, están la identidad celular, la viabilidad y tasa de muerte celular, la densidad celular al momento de recolectar el medio, los pasos previos al procesamiento y almacenamiento de la muestra, y el control de las vesículas extracelulares derivadas del suero. Cuando el medio se suplemente con suero, se debe indicar si este se usó o no en la obtención de las vesículas extracelulares y, si fue así, cómo se controlaron y eliminaron aquellas derivadas del suero o el lavado del cultivo entre cambios de medio.

A partir de los talleres de la ISEV y la creación en el 2017 de grupos de trabajo en vesículas extracelulares de fluidos biológicos específicos, recientemente el grupo enfocado en las vesículas extracelulares derivadas de sangre reportó los resultados de una encuesta que permite plantear una hoja de ruta para su recolección, manejo y almacenamiento, y resaltó la importancia de medir la calidad de la muestra antes del aislamiento y análisis de vesículas extracelulares ^15^.

## Aislamiento y purificación de vesículas extracelulares

Al trabajar con vesículas extracelulares, es ineludible reconocer que los métodos actuales de aislamiento solo pueden enriquecer un determinado subtipo, sin purificarlo o aislarlo completamente. Así, en función del protocolo que se siga, las preparaciones presentarán algún grado de heterogeneidad y una potencial contaminación con otras vesículas extracelulares y su contenido o con otros materiales diferentes, como proteínas, metabolitos y ácidos nucleicos libres ^7^^,^^66^^,^^67^. Por otro lado, actualmente no existe un estándar de referencia contra el cual puedan evaluarse las otras técnicas, ya que cada una se basa en principios distintos, con pros y contras, y permiten separar vesículas con diverso desempeño ^7^.

Considerando la capacidad de recuperación de vesículas y su especificidad, las técnicas de separación se pueden clasificar en:

1. técnicas de alta recuperación y baja especificidad, que permiten obtener una gran cantidad de material extracelular, incluyendo vesículas, proteínas y metabolitos presentes en la matriz, lo que se considera un secretoma concentrado parcial o completo;
2. técnicas de recuperación y especificidad intermedias, que permiten recuperar una población mixta de vesículas que puede estar contaminada con componentes de la matriz según los pasos de lavado empleados, y
3. técnicas de baja recuperación y alta especificidad, con las que se puede separar uno o más subtipos de vesículas en función de su tamaño, su densidad de flotación o densidad granular, sus moléculas de superficie y propiedades biofísicas como la carga superficial, con un menor contenido de componentes no vesiculares, pero en menor cantidad, principalmente porque se obtiene una población de vesículas extracelulares más específica (cuadro 4). Infortunadamente, aún no existe una técnica que satisfaga plenamente los criterios de alta recuperación y alta especificidad ^7^.

Los métodos basados en la centrifugación han sido los más usados en el campo ^10^. No obstante, presentan limitaciones como, que se aisle concomitantemente material no exosómico (incluidas las proteínas presentes en la matriz), que se produzcan daños estructurales en la membrana vesicular y que existan parámetros no estandarizados que conduzcan a variabilidad cualitativa y cuantitativa ^66^. Se ha visto que factores como el tipo de rotor, la fuerza *g* o fuerza centrífuga relativa *(Relative Centrifugal Force)* y los tiempos de centrifugación, afectan significativamente el proceso de separación de las vesículas extracelulares y, por lo tanto, la pureza y el rendimiento obtenidos, a tal grado, que es posible que los protocolos de aislamiento basados en centrifugación diferencial comúnmente recomendados no estén del todo optimizados ^12^.

La práctica de usar un protocolo de centrifugación común con distintos rotores, con frecuencia conduce a resultados diferentes e inadecuados, considerando que el ajuste de la duración de la centrifugación de acuerdo con los factores K del rotor es improcedente para rotores de "ángulo fijo" ^74^. Además, se ha visto que subtipos de vesículas extracelulares sedimentados a la misma velocidad muestran densidades de flotación diferentes. Empero, los protocolos de flotación en gradiente de yodixanol no pueden separar vesículas medianas de las pequeñas si no han sido separadas primero con otro método como la centrifugación ^69^.

Por lo tanto, si se quieren aislar subtipos discretos, se debe recurrir a una combinación de técnicas de separación ortogonales. En el caso de los estuches comerciales y de algunos protocolos, vesículas pueden estar potencialmente unidas o mezcladas con componentes introducidos, como anticuerpos, perlas, polímeros, etc. En el caso de ciertas técnicas de precipitación, es necesario considerar su especificidad y los potenciales efectos sobre las vesículas aisladas, lo que cobra relevancia cuando se pretende hacer experimentos funcionales con las vesículas extracelulares aisladas, ya que se pueden presentar artefactos, se requieren pasos adicionales de limpieza y es necesario contar con los controles adecuados acordes con la estrategia empleada ^7^.

Como ya se mencionó, la selección de las técnicas de aislamiento y caracterización dependerá del tipo de muestra, el volumen disponible del material de partida y las aplicaciones posteriores. El aislamiento de vesículas extracelulares de fluidos biológicos complejos suele requerir más pasos y estrategias combinadas que los necesarios en el caso de medios condicionados por cultivos celulares. De forma similar, los análisis proteómicos requieren un mayor control sobre la muestra y su calidad que los análisis por citometría de flujo o qPCR.

Se han desarrollado técnicas de aislamiento promisorias que superan a las tradicionales, como las basadas en filtración de flujo tangencial *(Tangential Flow Filtration),* entre ellas, la técnica de fraccionamiento en flujo de campo de flujo *(Flow Field-Flow Fractionation),* con la que se separaron vesículas extracelulares urinarias de tipo exosoma y se analizó su lipidoma en un estudio sobre cáncer de próstata ^75^, y el fraccionamiento en flujo mediante campo de flujo asimétrico (AF4, *Asymmetrical Flow Field Flow Fractionaltion),* con el cual se aislaron subtipos de vesículas extracelulares pequeñas de tipo exosoma y nanopartículas que luego fueron caracterizadas con base en su perfil proteómico ^11^.

Otra tecnología emergente es la de las plataformas basadas en microfluidos o chips de microfluidos, con las cuales se avizora el aislamiento, la cuantificación y caracterización de exosomas de forma automatizada ^76^. Estas cuentan con múltiples aproximaciones para analizar vesículas y otros componentes extracelulares, y permitirían avanzar en la investigación y posterior empleo de biopsias líquidas aplicadas al diagnóstico de enfermedades, pues reducen la intervención manual, los tiempos de ensayo, y la cantidad de muestra y reactivos requeridos; además, se aumenta la sensibilidad, lo cual posibilita el empleo de vesículas extracelulares en el diagnóstico y aporta al desarrollo de la medicina personalizada ^72^^,^^77^.

Aunque las guías MISEV se centran únicamente en técnicas de amplio uso, la del 2018 contempla nuevas técnicas desarrolladas o aplicadas en el aislamiento de vesículas extracelulares, las cuales deben verificarse a partir de la información sobre la caracterización de las obtenidas con ellas para demostrar su grado de éxito con respecto a las técnicas clásicas, como la ultracentrifugación diferencial y el gradiente de densidad. Estas tecnologías son rápidas, eficientes y permiten obtener vesículas extracelulares más puras y homogéneas que las técnicas clásicas; sin embargo, la mayor limitación actual reside en la complejidad de los dispositivos, su costo y poca disponibilidad. En el futuro, con el avance de la tecnología y el aumento de la oferta en el mercado, pueden llegar a convertirse en herramientas accesibles y de amplio uso, como fue el caso de la citometría de flujo para la clasificación de células activadas por fluorescencia FACS *(Fluorescence-Activated Cell Sorting).*

En definitiva, es posible obtener poblaciones enriquecidas en un tipo particular de vesícula extracelular, pero debe tenerse claro que no es posible aislar un subtipo con una homogeneidad o pureza del 100 % y un nivel de recuperación adecuado para desarrollar diferentes análisis y ensayos. Los investigadores deben ser conscientes del grado de heterogeneidad en la población de vesículas extracelulares obtenida, cuya posible composición y potenciales contaminantes dependerán de las condiciones experimentales y detalles del protocolo utilizado, incluido el cultivo y la obtención de vesículas extracelulares, la viabilidad celular, los pasos previos de separación, los pasos finales de lavado, etc.

## Caracterización de las vesículas extracelulares

Como se aprecia en el cuadro 4, las guías MISEV incluyen: 1) la descripción cuantitativa tanto de la fuente de vesículas extracelulares como de su preparación, 2) la caracterización de la forma general de las vesículas extracelulares, y 3) su caracterización individual.

Para la cuantificación de la fuente, se debe indicar el número de células cultivadas y los intervalos de recolección (cuando corresponda), el volumen total del biofluido de partida o el peso, volumen y tamaño del tejido en el momento de la recolección ^14^. Sin embargo, los parámetros apropiados varían según la fuente; así, para la orina, el volumen no sería significativo y se pueden informar otros parámetros, como la concentración de creatinina, siendo este también un parámetro para evaluar la pureza del aislamiento de vesículas extracelulares con respecto al fluido de origen.

En cuanto a la cuantificación de las vesículas extracelulares, se sugiere dar un valor global, pues no existe un único método de cuantificación perfecto, ya que ni el número total de partículas ni el de proteínas o de lípidos está asociado exclusivamente con las vesículas extracelulares: las proteínas también son solubles, las partículas pueden ser agregados de proteínas y los lípidos también están presentes en las lipoproteínas. Así, en la cuantificación se deben informar las relaciones de proteínas: partículas, lípidos: partículas o lípidos: proteínas, así como las estimaciones de cuantificación global como una medida de pureza.

En el caso de la orina, mientras que la creatinina se ha convertido en el factor de normalización habitual para los biomarcadores urinarios en fase líquida, en un estudio se vio que el número de vesículas extracelulares pequeñas y la proteína Tsg101 transportada en estas sirven para normalizar la variación circadiana cuando se prueban biomarcadores candidato en vesículas extracelulares pequeñas; estos dos parámetros, entonces, se han propuesto como factores de normalización viables para biomarcadores en vesículas extracelulares pequeñas. Este aspecto de la caracterización es vital, ya que una correcta normalización del número de vesículas es uno de los requisitos para poder trasladar al contexto clínico los biomarcadores candidatos encontrados en las vesículas extracelulares de orina descritas para enfermedades renales ^78^.

En la caracterización general, inicialmente se establecían cuatro categorías de proteínas: 1) extracelulares unidas a lípidos o transmembrana, 2) citosólicas, 3) intracelulares ausentes o contenidas en baja cantidad en vesículas extracelulares-exosomas, pero presentes en otros tipos de vesículas extracelulares, y 4) extracelulares asociadas con estas vesículas, de las cuales debía cuantificarse al menos una proteína de las categorías 1, 2 y 3 ^7^. Gracias al avance en el conocimiento de los diferentes subtipos de vesículas extracelulares existentes y el análisis de su contenido proteico, en las guías MISEV 2018 se propusieron nuevas categorías de proteínas para evaluar en cualquier preparación de vesículas extracelulares: 1) marcadores proteicos positivos de vesículas extracelulares asociados a la membrana y al citosol, 2) marcadores proteicos negativos como control de pureza, 3) marcadores específicos de un subtipo de vesículas extracelulares, y 4) proteínas extracelulares solubles con actividades funcionales.

Conscientes de que la muestra obtenida usualmente es limitada, no es necesaria la evaluación de estos marcadores en cada tanda de aislamiento, pero sí, por lo menos, cada vez que se modifiquen las condiciones preanalíticas o de aislamiento de las vesículas extracelulares. Esta combinación de marcadores es necesaria para demostrar la presencia de vesículas de membrana intactas en vez de fragmentos de membrana. Los controles de pureza incluyen proteínas que se encuentran como contaminantes aislados concomitantemente, los cuales dependerán de la fuente biológica, ya sean lipoproteínas y materiales derivados del suero o los presentes en la orina como la creatinina. También, se recomienda evaluar proteínas presentes en compartimentos subcelulares distintos de la membrana plasmática y los endosomas, como las mitocondrias, que pueden estar presentes en ciertos subtipos de vesículas extracelulares. Cuando se ha identificado un factor soluble funcional en las vesículas extracelulares, como citocinas, factores de crecimiento y componentes de la matriz extracelular, debe determinarse su modo de asociación con dichas vesículas o topología ^14^. La topología de los componentes asociados a las vesículas extracelulares es una característica nueva introducida en las guías MISEV 2018 que indica si estas son de la luz o superficiales.

Para la caracterización de las vesículas individuales, se deben emplear técnicas complementarias u ortogonales, entre ellas:

1. imágenes de alta resolución de vesículas extracelulares individuales mediante microscopía electrónica y técnicas relacionadas, microscopía de barrido por sonda (SPM), incluida la microscopía de fuerza atómica (AFM) o microscopía de superresolución, y
2. análisis de partículas individuales que estiman características biofísicas de las vesículas extracelulares como:

a. la medición del tamaño mediante sensor de fuerza resistivo o por propiedades de dispersión de luz mediante análisis de seguimiento de nanopartículas (NTA), citometría de flujo de alta resolución, detector de dispersión de luz multiangular acoplada a AF4, etc.,
a. propiedades de fluorescencia mediante espectroscopía de correlación de fluorescencia (FCS) o citometría de flujo de alta resolución, y
a. composición química medida por espectroscopía Raman.

Cada una de estas técnicas tiene sus limitaciones y no todas funcionan igual con todos los tipos de vesículas extracelulares: las muy grandes (> 400 nm) y las muy pequeñas (<50 nm) no se pueden cuantificar bien por NTA, y las pequeñas no son fáciles de detectar por los citómetros de flujo más comunes. Algunas vesículas grandes y agregados de vesículas pequeñas se pueden monitorear mediante microscopía de luz y fluorescencia; las vesículas por debajo del límite de refracción o de la resolución de un microscopio se pueden detectar por fluorescencia, pero no se puede obtener información estructural ni distinguir una vesícula individual de un agregado de vesículas pequeñas únicamente con base en detalles estructurales ^14^. Además de las limitaciones de tamaño del NTA, los grandes agregados de proteínas pueden confundirse con vesículas durante el análisis, sobreestimando la concentración de vesículas ^13^.

En consecuencia, la cuantificación puede verse sesgada por la medición de partículas contaminantes presentes en el solvente y por agregados de proteínas que provengan de la muestra y tengan un comportamiento similar al de las vesículas; por esto se deben usar controles adecuados, incluidos el solvente y medios no condicionados, y asegurar la limpieza de la muestra. Para superar dichos problemas, algunos investigadores han combinado estas técnicas con otras metodologías, por ejemplo, la espectroscopia Raman y la marcación fluorescente. Paralelamente, el desarrollo de citómetros de flujo de nueva generación equipados con detectores de partículas pequeñas ha permitido analizar directamente las vesículas extracelulares; sin embargo, no siempre se da una validación adecuada de estos análisis, especialmente para confirmar que se analizan vesículas individuales en lugar de agregados de vesículas extracelulares ^10^.

A pesar de las dificultades y retos mencionados, el desarrollo de estudios proteómicos ha permitido avanzar en el conocimiento, y proponer y validar marcadores para diversos subtipos de vesículas extracelulares, incluidas las grandes, las medianas y las pequeñas obtenidas por ultracentrifugación y según el subtipo de las pequeñas. Es claro que las proteínas consideradas marcadores de exosomas no deberían usarse como marcadores de una población vesicular específica, sino como proteínas enriquecidas en estas. Con base en el análisis proteómico cuantitativo extensivo desarrollado por Kowal, *et al.,* los autores propusieron una serie de marcadores proteicos específicos y validados para vesículas extracelulares pequeñas y exosomas, definiendo cinco categorías de proteínas con diferente abundancia relativa en distintos tipos de estas vesículas, así como dos posibles flujos de trabajo para un análisis exhaustivo de sus mezclas.

Como ya se mencionó, se encontraron proteínas marcadoras y asociadas a exosomas en poblaciones de vesículas extracelulares pequeñas CD9+ que no corresponden a exosomas. Si bien los marcadores que se pensaba que eran exclusivos de exosomas realmente eran genéricos para vesículas pequeñas, se ha visto que algunos marcadores proteicos son únicos de una subclase de las pequeñas que cargan tetraspaninas específicas ^69^. Los autores proponen la tetraspanina CD63 para aislar una población discreta de exosomas a partir de vesículas extracelulares pequeñas obtenidas a 100*g;* sin embargo, es necesario considerar que la CD63 también se encontró en vesículas grandes, razón por la cual se debe iniciar el protocolo con su remoción, y, además, no todos los tipos celulares expresan CD63 o secretan vesículas extracelulares con CD63. Así, para cada tipo celular se debe establecer la presencia y la combinación de marcadores exosomales adecuados para la caracterización vesicular.

En cuanto al análisis del ARN contenido en las vesículas extracelulares (EV-RNA), en el taller EV-RNA de la ISEV desarrollado en el 2015, se discutieron varios desafíos técnicos como la estandarización de los métodos de aislamiento, la optimización de metodologías para poder aislar y caracterizar las mínimas cantidades de ARN que se halla en las VE, y el desarrollo de aproximaciones para demostrar la transferencia funcional de EV-RNA en vivo. La publicación de los resultados evidencia el conocimiento incompleto en torno a la naturaleza de los EV-RNA y a cómo estudiarlos de manera efectiva y confiable, lo que impide la implementación de estándares de referencia en la investigación del EV-RNA; los autores piden precaución en la interpretación de los datos obtenidos, creando conciencia de las posibilidades y limitaciones de las estrategias utilizadas en el momento para investigar EV-RNA ^79^.

## Estudios funcionales con vesículas extracelulares

La comunicación celular requiere de mediadores solubles o moléculas de señalización, las cuales pueden ser secretadas por la célula que genera la señal al espacio extracelular por exocitosis, liberadas por difusión a través de la membrana plasmática, o expuestas al espacio extracelular mientras permanecen unidas a la superficie celular ^1^. Otra opción es su secreción y transporte a través de las vesículas extracelulares. La secreción y la comunicación mediadas por vesículas extracelulares constituyen un fenómeno evolutivo conservado; por lo tanto, se acepta la hipótesis según la cual estos son mensajeros celulares en el contexto de la comunicación celular, con funciones en la célula receptora gracias a la transferencia de proteínas, lípidos y ARN ^34^^,^^80^.

Sin embargo, algunas veces se hacen afirmaciones difíciles de respaldar experimentalmente. Una correcta categorización de los diversos tipos de vesículas extracelulares -acorde con su definición oficial- no solo es importante en sí misma, sino también, para poder establecer y precisar un rol funcional para cada subtipo de vesícula. Aunque se cree que distintas poblaciones vesiculares podrían generar efectos funcionales diferentes, inicialmente, la actividad biológica observada se atribuía exclusivamente a los exosomas, sin una adecuada caracterización ni confirmación de su origen endosómico, y no se consideraba la presencia de otras vesículas extracelulares y contaminantes en los preparados. Las recomendaciones de la MISEV 2018 buscan evitar artefactos e interpretaciones excesivas al analizar las funciones de estas vesículas y atribuir una actividad funcional a una vesícula en general o a un subtipo específico, como los exosomas.

En este sentido, la ISEV considera que no se debe atribuir una función específica a las vesículas extracelulares o a alguno de sus subtipos solamente con su descripción en una preparación heterogénea y potencialmente contaminada, aspecto importante en este tipo de estudios por los problemas ya analizados ^14^. Por lo tanto, según cuál sea la pregunta que se pretende contestar, o el problema que se quiere resolver, podría no ser tan relevante o factible experimentalmente asignar una función dada a un tipo específico de vesícula extracelular en lugar de estudiar la actividad biológica de un conjunto de vesículas derivadas de una condición *in vivo* específica, y comparar las derivadas de diferentes condiciones fisiopatológicas. Los investigadores deben ser conscientes de esto en el momento de sacar conclusiones para evitar la asociación de una actividad biológica con un tipo particular de vesícula extracelular, sin antes haber analizado y confirmado su identidad y pureza.

Las guías MISEV 2014 presentaban múltiples orientaciones para establecer si una actividad biológica estaba asociada o no con las vesículas extracelulares, específicamente con los exosomas ^7^. En las del 2018, estas evolucionaron y se ampliaron los aspectos por considerar sin centrarse en un tipo particular de vesícula extracelular. Para diferenciar las funciones específicas de los distintos tipos de vesículas extracelulares, es necesario establecer las contribuciones relativas de cada fracción vesicular en la actividad total, por ejemplo, las de las pequeñas frente a las grandes, e incluir como controles el medio o fluido original y reducido de las vesículas.

Esto es posible mediante la combinación de técnicas ortogonales de aislamiento. Así, la ISEV recomienda hacer las pruebas funcionales después de separaciones rigurosas, y comparar las fracciones de vesículas extracelulares y aquellas diferentes a estas para determinar qué proporción de la actividad está asociada con cada fracción. Si la actividad se asocia principalmente con las vesículas extracelulares, esta actividad debería reducirse al removerlas, si no es que desaparece. En este punto se habla de un medio o fluido con reducción o pocas vesículas extracelulares, y no libre de ellas, ya que no siempre es posible su remoción total del fluido o del sobrenadante. Esta separación refinada debe realizarse, al menos, para un conjunto de réplicas biológicas, aunque no necesariamente de manera sistemática después.

Las indicaciones de las guías MISEV incluyen los siguientes pasos:

1. normalizar la cantidad usada de vesículas extracelulares para llevar a cabo estudios funcionales comparativos, teniendo en cuenta las recomendaciones para la cuantificación;
2. demostrar que la actividad se observa en ausencia de contacto directo de célula a célula;
3. demostrar que la actividad se asocia predominantemente con estas vesículas en lugar de mediadores solubles;
4. demostrar la asociación específica de la actividad con vesículas extracelulares y no con componentes aislados simultáneamente y, según sea el caso, y
5. determinar si una función es específica de los exosomas, en comparación con otras vesículas pequeñas.

También, es posible atribuir efectos particulares mediados por vesículas extracelulares a componentes específicos de vesículas extracelulares y a vesículas extracelulares de un origen celular dado, cada uno con consideraciones para tener en cuenta. antes de concluir y asignar funciones específicas a un tipo vesicular en particular ^14^. En las guías MISEV 2018, se presentan ejemplos de protocolos para demostrar los puntos 1 a 4. En cuanto al punto 5, existen ciertas limitaciones técnicas que impiden concluir que los exosomas tengan funciones específicas en comparación con otras vesículas extracelulares. Gracias a los estudios más recientes en que se separan los múltiples subtipos vesiculares, ahora es claro que estos pueden presentar actividades funcionales tan importantes como las atribuidas a los exosomas.

La definición de los marcadores apropiados para describir distintas poblaciones de vesículas extracelulares permite, no solo caracterizar las obtenidas y estudiar su efecto biológico, sino también, investigar la maquinaria molecular específica requerida para su biogénesis y secreción. Teniendo en cuenta que estas vesículas se definen con base en su ruta de biogénesis y secreción, es importante y necesario estudiarlas y analizarlas. Además, la comprensión de estos mecanismos permite entender las funciones fisiológicas y patológicas de las vesículas extracelulares. Las estrategias de análisis incluyen la inhibición o promoción selectiva de la secreción de un tipo vesicular particular mediante fármacos, tratamientos y herramientas de inhibición génica. Sin embargo, los componentes de la maquinaria involucrada pueden ser compartidos por diversas vías, de tal forma que una estrategia puede afectar a más de un tipo de vía de ensamblaje y secreción y, por lo tanto, a más de un tipo de vesículas extracelulares, por lo que este otro aspecto debe considerarse y evaluarse sistemáticamente ^69^.

Para superar este escollo, se requieren estudios que identifiquen marcadores y blancos moleculares de rutas de biogénesis específicas y únicas, que afecten únicamente a un subtipo vesicular en particular, para poder evaluar el ensamblaje intracelular de las vesículas e identificar y aislar aquellas vesículas específicas después de que han salido de la célula. Afortunadamente, cada vez se cuenta con un mayor conocimiento y mejores herramientas para acelerar el desarrollo del campo y ampliar sus horizontes, y se espera que, en estudios futuros, se brinden herramientas más selectivas a la hora de promover o inhibir la secreción de un tipo particular de vesícula extracelular.

Por último, en los estudios funcionales con vesículas extracelulares, unas veces se presume y otras se investiga su captura por parte de la célula receptora. Sin embargo, son muchos los desafíos y los artefactos que pueden presentarse durante estos ensayos ^81^. La detección dentro de una célula de la señal de un tinte de marcado de vesícula extracelular no significa necesariamente que esta, o su carga, haya sido internalizada.

En la revisión de Mulcahy, *et al.,* se presentan varias cuestiones asociadas con los tintes fluorescentes de unión a membrana, los compuestos químicos y péptidos utilizados para inhibir la absorción de vesículas extracelulares y los artefactos que pueden aparecer, y se analiza cómo estos pueden afectar el comportamiento normal de las vesículas y las células receptoras. Aun así, la evidencia de que pueden ingresar a las células y entregar su carga, es cuantiosa ^81^. La guía MISEV 2018 no trae una recomendación clara al respecto, pero insiste en que los investigadores sean conscientes de estos problemas y consideren que cada par específico de célula donante-receptora de vesículas extracelulares puede comportarse de forma diferente ^14^.

Es necesario reconocer que no todos los biofluidos están disponibles en un volumen suficiente para aislar vesículas extracelulares y desarrollar múltiples análisis con cada muestra. En esos casos, las guías MISEV establecen que se pueden combinar varias muestras para establecer la fiabilidad de los métodos de separación y caracterización, antes de hacer análisis específicos o estudios funcionales con muestras individuales. En caso de que no se pueda llevar a cabo un análisis del método de separación y una caracterización adecuada, es responsabilidad de los autores advertir las interpretaciones alternativas a las que haya lugar, por ejemplo, que las vesículas extracelulares pueden contribuir, aunque no necesariamente de forma exclusiva, a un fenómeno observado o una firma molecular.

Las guías MISEV no son definitivas en algunos aspectos, al fin y al cabo son recomendaciones; no obstante, si las atendemos al plantear estudios y los autores ingresan los detalles experimentales de sus publicaciones a través de EV-TRACK, se podrán considerar y definir condiciones experimentales que, de otra forma, son pasadas por alto en la sección de materiales y métodos de los artículos, pero que influyen en la calidad de las preparaciones de vesículas extracelulares y la posibilidad de reproducir los resultados.

## Estado actual del estudio de vesículas extracelulares en Colombia

En Colombia, también ha habido un interés creciente en el estudio de los exosomas y otras vesículas extracelulares, como lo evidencia el aumento de publicaciones de investigadores con afiliaciones colombianas en los últimos cinco años. A partir de una revisión en PubMed utilizando los términos de búsqueda ("exosome" OR "microparticles" OR "extracellular vesicles") [Title/Abstract] AND "Colombia" [All Fields] (junio,2020), se encontraron tres trabajos referentes a exosomas, siete de vesículas extracelulares y 26 de micropartículas, incluidas micropartículas de carácter no biológico. Se hizo una búsqueda similar en Scielo ^82^ y en Google Scholar ^83^. Dado que la información que se buscaba debía ser de investigadores afiliados a una institución colombiana con publicaciones en el área de las vesículas extracelulares o exosomas, en esta revisión se excluyeron los estudios publicados por colombianos con afiliación en instituciones extranjeras.

Se encontraron artículos de revisión con la participación de investigadores afiliados a instituciones colombianas como coautores, los cuales se centran en exosomas derivados de células madre mesenquimales y su potencial terapéutico ^17^, así como artículos de revisión con autoría enteramente colombiana sobre micropartículas como moduladores de la reacción autoinmunitaria ^18^ y artículos originales con investigadores afiliados a instituciones colombianas entre los coautores ^19^. El grupo de Castaño, *et al.,* ha adelantado estudios sobre micropartículas formadoras de complejos inmunitarios, su papel durante la activación de monocitos ^20^^)^ y la diferenciación a macrófagos ^21^ en pacientes con lupus eritematoso sistémico y artritis reumatoide, así como sobre vesículas extracelulares circulantes en estos últimos y su efecto en la inducción de citocinas proinflamatorias y en la activación de fagocitos mononucleares ^22^. También, se han hecho estudios sobre la captura por células endoteliales de micropartículas derivadas de pacientes con estas dos enfermedades ^23^^)^ en los que las vesículas extracelulares fueron aisladas por ultracentrifugación diferencial y caracterizadas mediante citometría de flujo multiparamétrica y microscopía STEM.

En un trabajo riguroso y detallado, se evaluó la liberación de vesículas extracelulares por células epiteliales intestinales Caco-2 infectadas con rotavirus, las cuales se concentraron a 100.000g y se separaron parcialmente mediante gradientes de sacarosa; se obtuvieron poblaciones que expresaban marcadores de cuerpos apoptóticos y de exosomas ^24^. Asimismo, se han publicado estudios de vesículas extracelulares aisladas con el estuche comercial ExoQuick^TM^: uno en el que se evaluaron en saliva tetraspaninas relacionadas con "exosomas" aislados con este estuche y caracterizados por TEM y ELISA para CD9 y CD81 ^25^, y otro sobre el papel de vesículas extracelulares derivadas de neuronas en la diferenciación neural de células madre derivadas de adipocitos ADSC, aisladas con el mismo estuche y caracterizadas por TEM, NTA y marcadores de VE (Alix, CD63, CD9, HSP70, citocromo, calnexina, COX IV), y marcadas con fluorescencia para confirmar su transferencia durante los análisis funcionales ^26^. En estas dos últimas publicaciones se puede ver la heterogeneidad en el uso de los términos "exosomas" y "vesículas extracelulares", aun cuando se emplea el mismo tipo de aislamiento, lo que evidencia la observancia de las guías MISEV por parte de Roballo, *et al.*^26^.

Recientemente, Velandia-Romero, *et al.,* estudiaron vesículas extracelulares derivadas de macrófagos infectados con el virus del dengue y su efecto biológico en células endoteliales. Aislaron vesículas extracelulares de sobrenadantes de cultivo por ultracentrifugación, especificando los detalles experimentales y el rotor empleado, y se caracterizaron como "exosomas" y "cuerpos apoptóticos" mediante la determinación de la densidad por flotación y microscopía TEM y, posteriormente, inmunoprecipitación con el estuche comercial Exo-Flow™ (Anti CD63); los autores verificaron la presencia de marcadores proteicos y, por último, analizaron su contenido proteico y algunos miARN ^27^. Además, nombran de forma cuidadosa cada producto obtenido, diferenciando entre gránulos *(pellet),* vesículas extracelulares, exosomas y cuerpos apoptóticos, según la fase de procesamiento y la evidencia proporcionada por los marcadores proteicos.

En otros estudios se utiliza el término "vesículas membranales", objeto de estudio que corresponde a las vesículas extracelulares, y que incluyen aquellas liberadas por micobacterias ^28^ y las derivadas de células epiteliales intestinales Caco-2 infectadas con rotavirus que expresan marcadores de exosomas ^29^. El empleo de este término se dio antes de la publicación de las guías MISEV 2014 y de la incorporación del término "vesículas extracelulares" en la ontología génica y en los vocabularios controlados, lo que explica la falta de correspondencia con la terminología oficial. Así como estos, es posible que haya otros estudios sobre las vesículas extracelulares en los que se empleaba una terminología variada, pues en ese momento no se contaba con los términos oficiales.

Por fuera del alcance de este análisis están las publicaciones con autoría de colombianos pero sin afiliación a una institución colombiana, como es el caso Gil-Bona, *et al.*^84^ (C. M. Parra es la autora colombiana), Ospina-Prieto, *et al.*^85^, y de la Torre-Gómez, *et al.*^86^, los cuales no pueden ser consultados con las opciones de búsqueda vigentes en las bases de datos.

En cuanto a los proyectos en curso y otros ya finalizados, en el repositorio principal CENDOC del Ministerio de Ciencias, no se registran en el momento proyectos con títulos que incluyan las palabras 'VE, exosoma o vesículas extracelulares, ni en español ni en inglés ^87^. En el buscador del Ministerio de Ciencias, entre los proyectos SIGP, se registran tres sobre: la participación de los exosomas producidos por macrófagos en la activación endotelial y la dispersión del virus del dengue-2; los inmunomoduladores asociados con la infección por rotavirus, y la interrelación entre la terapia antitumoral clásica y los adyuvantes naturales en la inducción de una reacción antitumoral, pero ninguno con el término exacto de "vesículas extracelulares" ^88^.

En cuanto a los productos de proyectos, se registran siete trabajos con el término "exosomas", uno con el término "exosoma", y tres con el término exacto "vesículas extracelulares" ^89^, entre tesis de pregrado y maestría e informes finales de investigación, los cuales incluyen estudios relacionados con cáncer, células dendríticas infectadas por *Leishmania,* virus del síndrome respiratorio y reproductivo en calostro porcino, enfermedad periodontal, células mesenquimales del estroma derivadas de medula ósea, macrófagos humanos infectados con virus del dengue, así como un informe de la estancia posdoctoral de la autora de esta actualización en el Grupo de Fisiología Molecular del Instituto Nacional de Salud, titulado (Novoa-Herrán S.S, Gómez-Grosso L.A. "Aislamiento y caracterización parcial de vesículas extracelulares tipo exosomas de sobrenadantes de cultivos de células de melanoma expuestas a doxorrubicina y su efecto sobre la viabilidad y acortamiento de cardiomiocitos aislados", disponible en https://sba.minciencias.gov.co/Buscador_Productos/busqueda?q=Aislamiento+y+caracte rizaci%C3%B3n+parcial+de+ves%C3%ADculas+extracelulares+tipo+exosomas+de+sobrenadantes+de+cultivos+de+c%C3%A9lulas&pagenum=1&start=0).

Por otra parte, en noviembre del 2019, en Bogotá, se desarrolló un minisimposio sobre vesículas extracelulares en biomedicina organizado por el Instituto Distrital de Ciencia, Biotecnología e Innovación en Salud - IDCBIS, en el que se reunieron algunos investigadores colombianos con intereses y experiencia en torno al estudio de las vesículas extracelulares, quienes discutieron los últimos avances, las dificultades y los desafíos del campo, y manifestaron intenciones de trabajar conjuntamente.

Esto demuestra que hay interés y capacidad para estudiar las vesículas extracelulares en el país, aunque los estudios y trabajos desarrollados aún son pocos y su calidad varía en cuanto al cumplimiento de los parámetros de calidad y seguimiento de las recomendaciones de la ISEV.

El desconocimiento del campo de las vesículas extracelulares por parte de los investigadores y los editores de revistas no especializadas, puede conducir a imprecisiones en las publicaciones y las afirmaciones que presentan, pero, gracias al desarrollo de herramientas como la EV-TRACK, los investigadores se pueden familiarizar con los parámetros experimentales más relevantes. La plataforma EV-TRACK también permite monitorear el avance en los métodos de aislamiento y caracterización, y las guías experimentales requeridas, al tiempo que promueve el reporte estandarizado de parámetros experimentales con EV-METRIC, una herramienta valiosa para los investigadores y revisores en esta área.

En cuanto a las técnicas y tecnologías disponibles en Colombia, las publicaciones evidencian la fortaleza técnica de las universidades colombianas, pues cuentan con citómetros de flujo, ultracentrífugas, espectrómetros de masas y microscopios electrónicos con los que puede irse más allá que con las técnicas básicas de bioquímica y biología molecular. También, existen empresas colombianas que prestan servicios de análisis de muestras mediante rastreo de nanopartículas (NTA), dispersión de luz dinámica (DLS), y microscopía electrónica, lo cual permite dar cumplimiento a las guías MISEV y generar trabajos de calidad internacional.

## Análisis del contexto mundial y nacional

El estudio de las vesículas extracelulares no solo constituye un reto científico-técnico, sino un estímulo para los grupos que actualmente trabajan y proyectan trabajar en un área relativamente nueva y promisoria, donde un avance significativo se puede dar gracias a la integración de experiencias, capacidades y esfuerzos, y de las diversas técnicas y tecnologías disponibles en cada institución. La realidad nacional permite considerar la creación de una red de investigación en torno a las vesículas extracelulares que favorezca el desarrollo y el fortalecimiento de este campo en Colombia mediante el intercambio de experiencias, *know-how* y fortalezas técnicas, así como del planteamiento de proyectos interinstitucionales con acceso a equipos por medio de convenios que complementen y diversifiquen los análisis realizados y satisfagan los requisitos de las guías MISEV.

El campo científico, como sucede con ámbitos de otras actividades humanas, es orgánica, evoluciona y se autorregula, lo que es evidente en el desarrollo del campo de estudio de las vesículas extracelulares, que, aunque relativamente joven, ha tenido una evolución veloz que continúa sin tregua. Gracias a la creación de la ISEV y la publicación de las guías MISEV, las controversias que antes existían han disminuido, ya que existe un consenso de la comunidad científica, lo que evidencia que este es un campo de estudio establecido, aunque todavía tiene preguntas por resolver. La comunidad científica debe reconocer los retos, las limitaciones y los vacíos que aún enfrenta en este campo; al dilucidarlos, se podrán orientar las investigaciones futuras y las prioridades de financiación.

En cuanto a las cuestiones en las que aún no hay consenso, este debe construirse por medio de la presentación de informes de experimentos bien controlados que se ajusten a los derroteros normativos establecidos por la ISEV. Un ejemplo es la necesidad de avanzar en la categorización y caracterización de las múltiples poblaciones de vesículas extracelulares secretadas por las células, y de obtener más marcadores adecuados para los diferentes tipos celulares, así como de establecer parámetros y marcadores claros para determinar la pureza de las preparaciones según el medio, el fluido de origen y el tipo celular. También, está pendiente el desarrollo de guías oficiales para el análisis del EV-RNA y su inclusión en las guías MISEV, la construcción de un consenso en torno a las recomendaciones adicionales para el estudio de la captura de vesículas extracelulares durante los ensayos funcionales, y un mayor acceso a técnicas y tecnologías de última generación que permitan aislar y caracterizar tipos vesiculares específicos.

Los avances en tecnologías como la de los citómetros de nano-flujo (nFCM), la combinación de técnicas de análisis de partículas con espectroscopía Raman y la marcación fluorescente, así como el desarrollo de las técnicas de fraccionamiento de flujo y de plataformas basadas en microfluidos, son el nuevo horizonte de trabajo. Las técnicas y tecnologías propias de otras áreas, como las de los nanomateriales, permitirían el análisis de las propiedades fisicoquímicas de las vesículas extracelulares, como uso del potencial Z, y la marcación de vesículas extracelulares con fluoróforos y metales; además, la combinación de sistemas de fluidos con técnicas de análisis como la espectrometría de masas, por ejemplo, pueden ampliar aún más los horizontes de trabajo. Estas nuevas oportunidades también son desafíos, pues se requiere estandarizar y homogenizar su empleo para garantizar la reproducibilidad de los resultados.

Para evitar resultados y conclusiones engañosas, los investigadores en este campo debemos ser conscientes de los alcances y las limitaciones de cada tecnología y cada técnica aplicada al análisis de vesículas, y los desafíos que encierra su estudio. Esto permitirá plantear mejores preguntas de investigación que puedan responderse con veracidad y rigor. Paralelamente, los investigadores debemos ser transparentes y reportar cabalmente los métodos y parámetros experimentales empleados, cumpliendo con las exigencias y recomendaciones de las guías MISEV. En este sentido, las bases de conocimiento como la EV-TRACK brindan herramientas a autores, revisores, editores y financiadores para poner en práctica las guías experimentales MISEV a partir del papel que le corresponde a cada científico.

A pesar de los desafíos técnicos, el estudio de las vesículas extracelulares es prometedor a nivel funcional. Además de ser un mecanismo de comunicación, modulación y regulación celular a distancia, las proteínas y el ARN transportados en exosomas y otras vesículas extracelulares pueden representar firmas moleculares de estados fisiológicos y patológicos de sus células de origen, lo que constituye una biopsia líquida, y una potencial herramienta diagnóstica y terapéutica con aplicaciones en cáncer y enfermedades autoinmunitarias y virales; de ahí la importancia y el interés en su estudio.

Las vesículas extracelulares se han propuesto como una ruta indirecta de infección viral, incluida la del SARS-CoV-2, y como una contribución a la fisiopatología de la Covid-19. Recientemente, se ha visto que las vesículas extracelulares derivadas de células epiteliales de pulmón A549 transfectadas con genes del SARS-CoV-2, transportaban ARN viral y fueron captadas por cardiomiocitos derivados de células madre pluripotentes inducidas (hiPSC-CMs) transfiriendo el ARN viral, lo que condujo a una regulación positiva de los genes relacionados con la inflamación en las hiPSC-CMs. Estos hallazgos sugieren que las vesículas extracelulares que transportan ARN del SARS-CoV-2 constituyen una ruta indirecta de entrada del ARN viral en los cardiomiocitos ^90^. Esta interacción entre las vesículas y el virus puede ser explotada para el desarrollo de vacunas, fármacos antivirales y terapias basadas en ellas, especialmente aquellas derivadas de células madre mesenquimales, para el manejo de la Covid-19 ^91^^,^^92^, ya sea que las vesículas extracelulares sean naturales, nanoseñuelos *(nano-decoy)* o estén cargadas de antivirales ^93^.

Los estudios ómicos de vesículas extracelulares enfocados en la búsqueda de biomarcadores, y la aplicación de técnicas como la espectrometría de masas para su detección en biopsias líquidas, contribuyen directamente al desarrollo e implementación de la medicina traslacional y personalizada de precisión ^94^, con avances como los logrados en el campo de la oncología ^95^^-^^97^. Un reciente informe enfocado en las aplicaciones de las vesículas extracelulares en diagnóstico y tratamiento, evidencia el aumento de los estudios relacionados con las vesículas extracelulares, patentes y subvenciones, así como de empresas emergentes con una gran interés en los exosomas ^9^. Esto alienta la investigación sobre las vesículas extracelulares para identificar nuevos biomarcadores de enfermedades, y abrir el camino al desarrollo de herramientas de diagnóstico adecuadas que puedan estratificar enfermedades y brindar un tratamiento personalizado a los pacientes.

## Recomendaciones y conclusiones

Esta actualización es un llamado a la comunidad científica nacional para que adopte la terminología oficial y siga las guías experimentales recomendadas por la comunidad científica internacional, las cuales contienen los requisitos para el estudio riguroso de las vesículas extracelulares. Se aspira a motivar a investigadores, revisores, editores, jurados y financiadores, a emplear y divulgar los términos y la nomenclatura oficiales, y a seguir las guías MISEV.

La expresión "vesícula extracelular" fue el primero usado, es el más preciso y claro, y es el término genérico y oficial, indexado en la ontología génica y en otros vocabularios controlados, y aprobado por la comunidad internacional asociada en la ISEV; además, puede usarse como una base para definir características adicionales de la población aislada según la caracterización realizada. Por otro parte, el término "exosomas" se refiere únicamente a las vesículas extracelulares de origen endosómico, y debe usarse con precisión y veracidad. Es necesario reconocer las limitaciones de las técnicas y tecnologías empleadas en el aislamiento, caracterización y desarrollo de los estudios funcionales, y utilizar los controles adecuados en cada caso. También, es importante tratar de definir el subtipo vesicular estudiado, especialmente cuando se le atribuye una función o una aplicación. Un trabajo reflexivo, concienzudo y cabal, con una definición clara de las vesículas extracelulares estudiadas, y que sea cauteloso en las afirmaciones y conclusiones, posibilitará el desarrollo de estudios con la calidad adecuada para el avance del campo.

Como un eco de las recomendaciones de la ISEV contenidas en las guías MISEV, esta actualización busca brindar orientación en cada fase de la investigación científica, desde el planteamiento de la pregunta de investigación, planeación y diseño experimental, hasta el análisis crítico de los resultados y su divulgación. Para esto, el consorcio de EV-TRACK recomienda la observancia de los parámetros EV-METRIC. Su empleo permitirá detectar limitaciones potenciales en los estudios de vesículas extracelulares y aumentar el rigor experimental, con el fin de que el campo pueda seguir creciendo y alcance todo su potencial.

Para concluir, en la presente actualización se plantea que el de las vesículas extracelulares es un campo científico consolidado, aunque aún en evolución, que cuenta con una terminología y guías oficiales, y se resalta el trabajo de instituciones como la ISEV, el desarrollo de las guías MISEV y la creación de recursos como la EV-TRACK. Aunque la clasificación de las vesículas extracelulares todavía está en desarrollo, se insiste en el uso correcto de los términos y la nomenclatura oficial. Se resumen las técnicas, recursos, requisitos y especificaciones de calidad disponibles en el campo, considerando las limitaciones, retos y oportunidades, y se brinda una visión de su futuro en el contexto mundial y el nacional, proponiendo perspectivas de trabajo para el avance del campo.
